# The *usage divide* of digital health technology in age-friendly home modifications: an ethnographic study among older adults in rural China

**DOI:** 10.3389/fpubh.2026.1735560

**Published:** 2026-02-09

**Authors:** Hong Zhang, Shuang Liang, Lin Wu, Yixin Wang, Lanyue Luo, Bin Peng, Xue Xiong, Liyu Chen, Qianying Jia, Tao Dai, Yuan Jia, Lily Dongxia Xiao, Liu Ren, Xiaoli Zhang, Jun Shen

**Affiliations:** 1 Department of Nursing, The First Affiliated Hospital of Chongqing Medical University, Chongqing, China; 2 Chongqing Nursing Vocational College, Chongqing, China; 3 Chongqing College of Architecture and Technology, Chongqing, China; 4 School of Public Health, Chongqing Medical University, Chongqing, China; 5 College of Nursing and Health Sciences, Flinders University, Adelaide, SA, Australia; 6 Chongqing University of Chinese Medicine, Chongqing, China; 7 The People's Hospital of Tongliang District, Chongqing, China

**Keywords:** age-friendly home modifications, aging in place, digital health technologies, ethnographic research, field study, older adults, rural areas, *usage divide*

## Abstract

**Objective:**

Digital health technologies, as an integral component of age-friendly home modifications in rural China, provide a promising pathway to address the challenges of population aging. However, a *usage divide* has emerged as a critical barrier, preventing older adults in these areas from fully benefiting from these technologies. This study aims to systematically examine this divide in the adoption of digital health technologies for age-friendly home modifications among older adults in rural China and to generate empirical evidence to inform policy and practice improvements.

**Methods:**

This study employed a focused ethnographic approach, informed by the constant comparative method of grounded theory. Intensive fieldwork was undertaken from June to December 2024. Data were generated through participant observation and semi-structured interviews and subsequently analyzed using thematic analysis.

**Results:**

Within the context of age-friendly home modifications among older adults in rural China, the *usage divide* in digital health technology emerged across four interrelated dimensions. The *skills divide* was manifested in cognitive misunderstandings of functional purposes, procedural barriers to execution, and inadequate problem-solving strategies. The *motivation divide* stemmed from intergenerational gaps in value perception, multiple manifestations of psychological resistance, and persistently low self-efficacy. The *support divide* was reflected in the formalization dilemma of formal training, systemic gaps in family support, and resource bottlenecks in community-based support. Finally, the *persistence divide* encompassed barriers to forming stable technology habits, difficulties in adapting to system updates, and the cumulative effects of repeated setbacks.

**Conclusion:**

Within the framework of age-friendly home modifications for older adults in rural China, the application of digital health technologies must transcend the narrow assumption that access alone guarantees use, shifting instead toward a comprehensive perspective that spans the continuum from access to effective utilization. The *skills divide* represents a fundamental barrier that undermines operational competence, while the *motivation divide* shapes willingness and readiness to engage with technology. The *support divide* provides the external conditions necessary to sustain both skills and motivation, and the *persistence divide* captures the temporal dynamics through which these dimensions evolve. Collectively, this multidimensional and dynamic *usage divide* explains why older adults in rural areas often fail to achieve effective use even after gaining access. It also reveals the mechanisms that determine whether technological availability is ultimately transformed into genuine empowerment. These findings contribute empirical evidence to refine rural age-friendly modification policies, narrow disparities in digital health technology use, and advance the dual goals of healthy aging and digital inclusion.

## 1 Introduction

Population aging has become one of the most significant social challenges of the 21st century. According to projections from the World Health Organization (WHO), the number of people aged 60 years and older is expected to reach 1.4 billion by 2030 and 2.1 billion by 2050, with nearly 80% residing in low-and middle-income countries (1). This demographic shift is reshaping long-term care systems worldwide and exerting unprecedented pressure on health resource allocation, social security arrangements, and models of care (2). The tension between traditional family-based care and the growing demands of modernization is particularly pronounced in resource-constrained rural areas, underscoring the urgent need for innovative and sustainable solutions (3).

As a hallmark of the Fourth Industrial Revolution, digital health technologies provide promising avenues to address the global challenges of population aging. By integrating the Internet of Things (IoT), artificial intelligence (AI), big data analytics, and telemedicine, these innovations enable real-time health monitoring, early warning, and timely intervention for older adults (4). Evidence suggests that digital health holds substantial potential to enhance quality of life, reduce healthcare expenditures, and delay functional decline among older populations (5). However, the transition from mere accessibility to effective use and tangible benefits is far from straightforward. Its success depends on multiple contextual factors, with these complexities being particularly pronounced among older adults in rural settings (6).

China, as one of the largest developing countries and home to the world’s biggest older adult population, is experiencing population aging under the distinctive conditions of getting old before getting rich and getting old before being well-prepared (7). According to *the Seventh National Population Census in China*, the number of people aged 60 years and older has reached 264 million, accounting for 18.7% of the national population (8). The challenge is even more acute in rural areas, where the older adult population has reached 121 million, representing 23.81% of the rural population, which is 7.99 percentage points higher than in urban areas (8). This inverted urban–rural aging pattern is closely tied to China’s dual social structure and has further marginalized rural older adults in their adoption of digital health technologies.

The rapid acceleration of industrialization and urbanization has brought profound social transformations to rural China. The large-scale outmigration of younger laborers has created widespread empty-nest households, severely weakening the traditional family-based system of elder care (9). Data from *the Fifth National Sample Survey on the Living Conditions of Urban and Rural Older Adults* indicate that the proportion of rural empty-nesters rose to 61.9% (10), compared with 37.9% in 2000 (11). The migration of adult children reduces the familial resources available for daily care and emotional support, and it is closely associated with heightened health risks, including heavier burdens of chronic disease, declining cognitive function, and more frequent depressive symptoms (12). At the same time, structural constraints in rural areas, including limited medical resources, inconvenient transportation, and restricted financial capacity, further exacerbate the health vulnerabilities of older adults and increase their risks of digital exclusion (7).

To address the challenges of rural population aging, the Chinese government has elevated “actively responding to population aging” to the level of a national strategy and established a multi-tiered care system in which family-based care functions as a supplementary option. Within this framework, age-friendly home modifications have emerged as a key initiative to improve the quality of home-based elder care. In July 2020, the Ministry of Civil Affairs, together with nine other government departments, issued joint guidance to accelerate the implementation of such projects. This policy incorporated “digitalized and intelligent elder care services” into the official modification standards, formally integrating digital health technologies as a central component of home modification efforts.

By the end of 2024, China had completed age-friendly home modifications for 2.08 million households of older adults living with special difficulties (13). These projects covered three main categories of digital health technologies (14). First, environmental sensing technologies, including fall detection sensors, smoke alarms, and gas leak detectors, were deployed to monitor home safety. Second, physiological monitoring technologies, such as wearable devices including smart wristbands, blood pressure monitors, and blood glucose meters, enabled continuous tracking of health indicators. Third, service-connection technologies, including telemedicine platforms, smart pillboxes, and one-touch call systems, were implemented to improve access to medical services. Although these technologies theoretically promise significant improvements in home safety, convenience, and quality of care, their actual effectiveness depends on the complex interplay of human, technological, and environmental factors, with disparities particularly evident among older adults in rural areas (15).

As a structural inequality of the information age, the *digital divide* has evolved from a simple gap in access to a complex, multidimensional challenge (16). The three-level theoretical framework of the digital divide explains this progression. The first level, the *access divide*, refers to the availability of physical devices and network connectivity. The second level, the *usage divide*, reflects disparities in skills, frequency, and diversity of technology use. The third level, the *outcome divide*, highlights differences in the actual benefits derived from technology. With the rapid expansion of rural network infrastructure and the implementation of age-friendly home modifications in China, the *access divide* has narrowed, whereas the *usage divide* has become increasingly salient, now constituting the main barrier preventing rural older adults from benefiting from digital health technologies (17).

The *usage divide* not only limits the realization of technological benefits but may also exacerbate a “Matthew effect” that intensifies health inequalities (18). Evidence indicates that even under equivalent access conditions, rural older adults lag behind their urban counterparts in the depth, breadth, and sustainability of technology use, owing to disparities in digital literacy, health literacy, social support, and cultural perceptions (19). This divide constrains their capacity to obtain digital health services and heightens the risk of further marginalization in the digital transformation, effectively creating a vulnerable subgroup within the digitally disadvantaged.

Given the *usage divide* in digital health technologies among older adults in rural China, existing research reveals three critical gaps that constrain a deeper understanding of technological inclusion in resource-limited settings:

First, urban bias and neglect of the *usage divide*. Most studies focus on urban populations in developed or developing countries, with little systematic examination of rural contexts, particularly the unique sociocultural setting of rural China (20). More importantly, many studies conflate “access” with “use,” overlooking the complex transition from mere availability to effective utilization (3).

Second, methodological dominance of quantitative approaches and the absence of user experience. Existing work relies heavily on questionnaires, randomized controlled trials, and other quantitative methods, emphasizing measurable outcomes such as adoption rates, frequency of use, and health indicators (21). While these studies provide valuable evidence of effectiveness, they fail to capture the dynamic processes and context-specific characteristics of technology use.

Third, a techno-deterministic perspective and the marginalization of cultural factors. Much of the literature treats technical features, such as ease of use and perceived usefulness, as the primary determinants of adoption, while reducing sociocultural elements to peripheral background variables (22). Such technology-centered frameworks neglect the formative influence of deeper factors, including cultural values, social networks, intergenerational relationships, and power structures, in shaping the *usage divide*.

As a contemporary extension of traditional ethnography, focused ethnography is particularly well suited to conducting in-depth investigations of specific issues within constrained time frames (23). It retains the contextual sensitivity and interpretive richness of ethnographic inquiry while improving efficiency, which has facilitated its wide application in health research (24). Accordingly, focused ethnography provides distinct methodological advantages for addressing the aforementioned research limitations and for deepening understanding of the *usage divide*.

Building on the above background and identified research gaps, this focused ethnographic study systematically investigates the *usage divide* in digital health technologies among older adults in rural China. The specific objectives are:

1. To reveal how the usage divide manifests in daily life, and to capture micro-level interactions and informal practices often overlooked in quantitative research.
2. To trace the dynamic processes through which the divide unfolds and to identify critical turning points from initial exposure to continued use or eventual abandonment.
3. To explain the sociocultural roots of the divide by examining how specific social relationships and cultural contexts shape technology use.

This study holds significance at both theoretical and practical levels. Theoretically, by concentrating on the *usage divide* as a core concept, it advances understanding of the second level of the *digital divide*. It moves beyond a simplistic binary of technology access versus non-access and reveals the complexities of actual technology use. Practically, the findings offer precise guidance for optimizing age-friendly home modification policies in rural China. They also contribute empirical evidence from a developing-country context to support efforts to narrow the *usage divide* and promote digital health equity.

## 2 Methods

### 2.1 Operational definition of the *usage divide*

Drawing on the three-level framework of the *digital divide* (16), this study defines the *usage divide* as the systematic disparity between rural older adults’ actual engagement with digital health technologies and the intended design parameters under the condition that physical access is available.

### 2.2 Study design

This study adopted a focused ethnographic approach to explore the *usage divide* in digital health technologies within the context of age-friendly home modifications among older adults in rural China. Focused ethnography is characterized by short-term but intensive fieldwork. It is particularly suited to examining how specific social phenomena unfold in everyday life (23). Given the health conditions of older adults, their geographical dispersion, and the limits of research resources, this method helped reduce participant burden while still generating rich and nuanced data on the *usage divide*.

The research design integrated the constant comparative method in grounded theory as developed by Glaser and Strauss (25). Fieldwork was organized into three intensive rounds, each lasting 4 weeks (June, September, and December 2024). Between rounds, two-month intervals were scheduled for preliminary analysis, theoretical reflection, and refinement of observation strategies.

The ethnographic process followed Spradley’s (26) cyclical framework. This framework comprises six interrelated stages: defining the problem and designing the study, entering the field and managing researcher roles, collecting primary data, recording and reporting, exiting the field, and conducting analysis and theory construction. Throughout the research, a reflective stance was maintained. Special attention was paid to how the researchers’ outsider position influenced the interpretation and understanding of the *usage divide*.

### 2.3 Setting and participants

#### 2.3.1 Research setting

Bishan District (106°02′-106°20′E, 29°17′-29°53′N) in Chongqing City served as the ethnographic field site. The rural aging rate in Bishan has reached 36.63% (27), which is higher than the national rural average of 23.81% (8). Rural settlements follow a “large dispersion, small aggregation” pattern, creating a natural context for comparing technology use across different community environments. As one of Chongqing’s first pilot areas for age-friendly home modifications, Bishan District has developed a relatively mature ecosystem of digital health technology applications.

Since 2020, Bishan District has completed age-friendly home modifications in 1,250 households. It has also established an integrated application system of digital health technologies that includes environmental sensing, physiological monitoring, and service connection. This stage of implementation allowed the study to concentrate on the *usage divide* rather than on the infrastructure-level *access divide*. In addition, the research team benefited from established collaborations with local civil affairs departments and grassroots organizations, which facilitated access to older adults with varying levels of technology use.

#### 2.3.2 Participant recruitment

Participants were recruited using purposive sampling combined with a maximum variation strategy (28). Eligible households were first identified through the age-friendly home modifications database maintained by the Bishan District Civil Affairs Bureau. Community staff at the grassroots level then assisted in contacting potential participants.

The core participants were rural older adults. Inclusion criteria were as follows: ① aged 60 years or above with local rural household registration; ② continuous residence at their current address for at least six months; ③ reliance on family-based care in the previous 12 months; ④ completion of age-friendly home modifications at least three months prior to recruitment; ⑤ installation of at least one digital health technology in the household, regardless of use; and ⑥ basic communication ability with informed consent provided. Exclusion criteria included: ① severe cognitive impairment; ② acute or terminal illness; ③ severe hearing or visual impairment not correctable by assistive devices; and ④ participation in a similar study within the past 3 months. Recruitment was guided by the need to ensure diversity in age, gender, education, functional status, living arrangements, housing type, health condition, and digital literacy.

To capture multiple perspectives on the *usage divide*, four groups of supplementary informants were also included: family caregivers, community staff, primary healthcare providers, and technical support personnel.

Sample size was determined according to the principle of theoretical saturation (29). Data collection and analysis were conducted concurrently, and recruitment ceased once new data no longer generated additional insights or attributes for the core categories. In total, 42 participants were included: 24 core participants (older adults from 24 independent households) and 18 supplementary informants.

### 2.4 Data collection

Between June and December 2024, data were collected through participatory observation and semi-structured interviews.

#### 2.4.1 Participatory observation and field notes

The researcher assumed the role of a *participant-as-observer*, engaging moderately in daily activities while conducting observations, with participants fully informed of the researcher’s identity. This role facilitated the development of trust and enabled access to insider perspectives, while maintaining the analytical distance for critical reflection. Observations focused on how the *usage divide* appeared and developed in daily life, using a progressive strategy that moved from external to internal and from surface to depth. Three sequential rounds of observation were conducted, each with a distinct but connected emphasis.

*Round 1* (*June 2024, Descriptive Observations*): This phase aimed to identify the surface characteristics of the *usage divide*. Key activities included mapping the spatial layouts of 24 households and marking the installation locations, accessibility, and visible usage traces (such as dust accumulation and signs of wear) of digital health devices; observing daily interaction, or the absence of interaction, between older adults and these technologies; recording time patterns and contextual triggers of device use or non-use; identifying environmental factors that influenced engagement with technology; and conducting a preliminary assessment of the severity of the *usage divide* across households.

*Round 2* (*September 2024, Focused Observation*): This round sought to explore the underlying mechanisms of the *usage divide*. Observations concentrated on the specific challenges and barriers encountered during device use; coping strategies adopted by older adults; the supportive or obstructive roles played by family members; differences between formal training and informal learning processes; the impact of the *usage divide* on older adults’ self-efficacy and social participation; and the identification of pivotal events that either promoted or hindered continued usage.

*Round 3* (*December 2024, Selective Observation*): This stage focused on validating the theoretical framework of the *usage divide*. Major tasks involved: verifying the typology developed in earlier stages through targeted observation; examining how cultural beliefs and other contextual factors shaped technology-use attitudes; analyzing the influence of the *usage divide* on family dynamics and community networks; identifying critical factors associated with successfully bridging the divide; and conducting member checking to ensure the credibility of the findings.

Each household was observed two to three times during each round, with sessions lasting between 2 and 4 h and scheduled at different times of day. A total of 186 observation sessions were conducted. A specially designed “Technology Usage Context Observation” protocol was applied to capture real-time interactions when older adults engaged with digital health technologies, such as measuring blood pressure or contacting family members. Observations recorded: operating procedures, encountered difficulties, emotional reactions, and strategies used to solve problems. All observations were systematically documented using Spradley’s (26) nine-dimensional framework: Space, Actor, Activity, Object, Act, Event, Time, Goal, and Feelings.

Field notes were organized into three categories, with close attention to the nuances of the *usage divide*: ① Descriptive notes recorded objective factual details related to the *usage divide*, including specific operational steps, types of errors, instances of seeking assistance, and moments when older adults abandoned usage (107 pages, approximately 110,000 words). ② Analytical notes captured initial theoretical reflections, identified various types of usage barriers, and explored the interrelationships among influencing factors (82 pages, approximately 90,000 words). ③ Reflective notes examined how the researcher’s own technical background and generational differences may have shaped the interpretation of older adults’ experiences (46 pages, approximately 70,000 words).

#### 2.4.2 Semi-structured interviews

The initial version of the interview guide was developed based on the Technology Acceptance Model (TAM) (30), the Unified Theory of Acceptance and Use of Technology (UTAUT) (31), and the “use” dimension of the *Digital Divide* framework (16). The final guide was refined through internal group discussions, expert consultations, and three pilot interviews. It encompassed the following dimensions: cognitive and functional understanding of technology, motivations and barriers to use, learning processes and support systems, user experience and emotional feedback, and intentions and behaviors related to continued use. The guide remained open-ended and flexible, allowing for ongoing adjustments informed by insights from field observations.

A total of 81 semi-structured in-depth interviews were conducted. These included longitudinal interviews with 24 older adults, each participating in at least three sessions designed to trace changes in their experiences of the *usage divide*. The first round focused on rapport building, the second on in-depth exploration, and the third on supplemental validation. Additionally, single-session interviews were conducted with 18 supplementary informants. A funnel-shaped questioning approach was adopted, beginning with open-ended descriptions of lived experience and gradually narrowing to specific manifestations and influencing factors of the *usage divide*. To enhance comprehension and encourage authentic responses, local dialects and everyday metaphors were incorporated. For instance, smart bracelets were described as electronic nannies, and telemedicine was explained as seeing the doctor through video.

All interviews were conducted either in participants’ homes or in quiet, designated spaces to promote a comfortable and natural conversational atmosphere. Each session lasted between 45 and 90 min (*m* = 52 min), and all were audio-recorded and supplemented with field notes. Transcription was completed within 24 h of each session and captured not only the verbal content but also non-verbal cues such as dialect expressions, tone, pauses, and emotional fluctuations. In total, the interviews yielded 73 h of audio recordings and 486 pages of transcript text.

### 2.5 Document analysis

Data were analyzed using Braun and Clarke (32) thematic analysis approach, combined with the constant comparison method from grounded theory (25). The analysis centered on the *usage divide* as the core focus and followed a six-step iterative process. First, the research team engaged in data familiarization by repeatedly reading transcripts and field notes, while also replaying audio recordings to capture non-verbal and paralinguistic cues. Preliminary memos were written to document emerging insights, and weekly immersion meetings were held to synthesize findings and refine analytical directions. Second, an inductive open coding strategy was applied using NVivo 12.0. Texts were analyzed line by line, and relevant segments were coded. A comprehensive codebook with clearly defined codes and examples was developed. Two researchers independently coded 20% of the dataset to assess intercoder consistency. Discrepancies were discussed and resolved through consensus, resulting in a unified coding framework. Third, initial codes were clustered into potential themes through pattern recognition, and relationships among codes were visualized using mind maps. Recurring patterns were identified, and a hierarchical thematic structure was developed that included major themes, subthemes, and minor themes. Memos documented the reasoning behind theme development. Fourth, themes were reviewed against the dataset to ensure internal homogeneity and external heterogeneity. Overlapping themes were merged, and heterogeneous themes were refined or split. A thematic diagram was created to illustrate the relationships among themes. Fifth, each theme was defined by clarifying its core meaning and analytical relevance. Representative quotes were selected to illustrate interpretation, and concise theme names were finalized through team discussions. Finally, the themes were integrated into a coherent theoretical narrative. Selected ethnographic excerpts were used to highlight key analytical insights, ensuring a balanced integration of empirical data and theoretical interpretation.

### 2.6 Rigor

To ensure research quality, the study adopted Lincoln and Guba’s (33) trustworthiness framework, incorporating strategies for credibility, dependability, transferability, and confirmability. Credibility was enhanced through several measures. Triangulation was used to examine whether different data sources on the *usage divide* showed consistent patterns. Long-term engagement in the field helped build a deeper understanding of the phenomenon. Member checks were conducted to ensure that the findings accurately reflected the real experiences of the participants. Dependability was supported by a clear audit trail. Each analytical step related to the *usage divide* was documented. Regular peer debriefings were held to review coding consistency. All versions of analytic memos were preserved to demonstrate how the theoretical framework evolved over time. Transferability was addressed by providing thick descriptions of the research setting, participant profiles, and the ways in which the *usage divide* appeared in real life. These details enable readers to judge whether the findings are applicable to other contexts. The study also acknowledged that the *usage divide* is context-dependent, which helped avoid overgeneralization. Confirmability was strengthened through reflective practices. The researchers regularly examined how their own backgrounds and positions might have influenced the interpretation of the *usage divide*. Clear links were maintained between the data, the analytic process, and the conclusions. In addition, external researchers who were not involved in data collection conducted independent analysis to validate the findings.

In this study, we adopted Clifford Geertz’s practice of “thick description” (34) to uncover the deeper layers of meaning within cultural and social phenomena. This approach prioritizes rich contextualization, facilitating a more comprehensive interpretation of participants’ experiences regarding the *usage divide*.

### 2.7 Ethical considerations

This study involving human participants were reviewed and approved by the Ethics Committee of the First Affiliated Hospital of Chongqing Medical University (Approval No.: 2023–035). All procedures strictly adhered to the principles of the Declaration of Helsinki and China’s Ethical Review Measures for Life Sciences and Medical Research Involving Human Beings (2023 Edition). At baseline, all participants signed written informed consent after being fully informed of the study’s purpose, potential risks and benefits, and what participation would involve. Before each observation, written consent was reconfirmed. The participants provided their written informed consent to participate in this study. During the entire data collection process, researchers continuously monitored the validity of participants’ verbal consent. Participants were explicitly reminded of their right to withdraw at any time, with assurances that doing so would not affect their access to services.

Privacy protection followed a three-tier protocol. At the data level, anonymization and desensitization of sensitive information were applied. At the technical level, encrypted storage and access control mechanisms were used. At the reporting level, details were moderately generalized to minimize the risk of indirect identification. In addition, local research assistants with at least 5 years of experience in rural geriatric care were involved to serve as cultural mediators and reduce potential bias in interpreting the *usage divide*. Throughout the study, participants’ autonomy, dignity, and well-being were consistently prioritized and safeguarded.

## 3 Results

### 3.1 Participant characteristics

A total of 42 individuals participated in this study. Among them were 24 rural older adults who had received age-friendly home modifications. In addition, 18 supplementary informants were included to provide contextual and professional perspectives. These consisted of 4 family caregivers, 5 community workers at the primary level, 6 healthcare providers from local medical institutions, and 3 technical support staff. The demographic and background characteristics of all participants are presented in Table 1.

**Table 1:** Characteristics of participants (*N* = 42).

Category	Characteristics		Categories	*n*	%
Older adults	Age (years)		60–69	8	33.3
			70–79	10	41.7
			≥80	6	25.0
	Gender		Male	15	62.5
			Female	9	37.5
	Living arrangement		Living alone (empty-nester)	11	45.8
			Living with spouse	8	33.3
			Living with children	5	20.9
	Residence type		Near township (≤5 km)	9	37.5
			Remote village (>5 km)	15	62.5
	Functional status		Independent in Activity of Daily Living (ADL)	5	20.8
			Mild dependence	10	41.7
			Moderate dependence	6	25.0
			Severe dependence	3	12.5
	Duration of technology use		3–6 months	6	25.0
			7–12 months	9	37.5
			>12 months	9	37.5
	Types of digital health technology configured		Environmental sensing	18	75.0
			Physiological monitoring	24	100.0
			Service connection	21	87.5
Supplementary informants	Family caregivers	Relationship to older adults	Spouse	1	25.0
			Adult child	3	75.0
		Caregiving duration (years)	<1	1	25.0
			1–3	2	50.0
			>3	1	25.0
	Community staff	Occupation	Village committee cadre	2	40.0
			Civil affairs specialist	1	20.0
			Community grid worker	2	40.0
		Work experience in older adults care (years)	<2	1	20.0
			2–5	3	60.0
			>5	1	20.0
	Primary healthcare providers	Professional title	General practitioner	3	50.0
			Nurse	2	33.3
			Public health worker	1	16.7
		Frequency of technology-assisted care	Weekly	4	66.7
			Monthly	2	33.3
	Technical support staff	Service duration in the region (months)	6–12	1	33.3
			>12	2	66.7
		Maintained technology types	Environmental sensing devices	3	100.0
			Physiological monitoring devices	2	66.7
			Service connection systems	3	100.0

### 3.2 Identified themes

**Figure 1:**
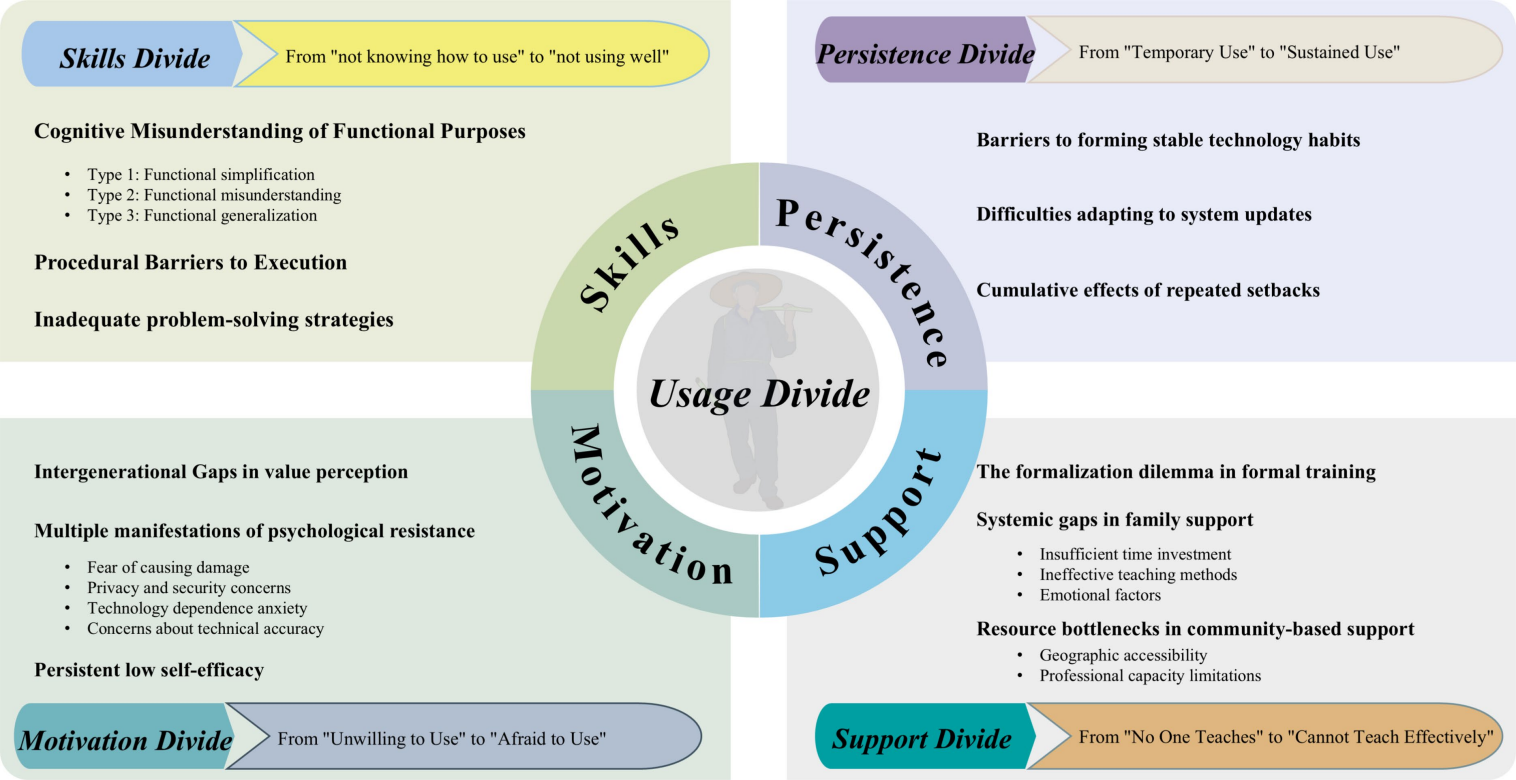
Relationships between the themes and their subthemes.

Through three rounds of intensive interval fieldwork and systematic thematic analysis, this study identified four core dimensions of the *usage divide* in digital health technology among rural older adults who had received age-friendly home modifications. These dimensions are the *Skills Divide, Motivation Divide, Support Divide*, and *Persistence Divide*. Rather than existing independently, they are closely interwoven and mutually reinforcing through everyday practices, collectively shaping the complex landscape of technology use. The relationships between themes and subthemes are illustrated in Figure 1.

#### 3.2.1 *Skills divide*: from “not knowing how to use” to “not using well”

As the fundamental dimension of the *usage divide*, the *skills divide* refers to the gap between the skills required to use digital health technologies and the actual capabilities of individuals or groups (35). This gap extends beyond operational proficiency to include limitations in functional understanding, contextual application, and adaptive responsiveness. Among older adults in rural China, digital literacy remains generally low; many can only manage basic tasks such as making phone calls or sending voice messages on WeChat (36). This pre-existing lack of skills further widens the disparity in effectively engaging with more complex digital health technologies.

##### 3.2.1.1 Sub theme 1: cognitive misunderstanding of functional purposes

Despite receiving standardized initial training, many rural older adults displayed systematic misunderstandings about the intended functions of digital health technologies. These stemmed from a mismatch between users’ existing cognitive frameworks and the operational logic of new technologies, and typically appeared in three recurring patterns.

*Type 1: Functional simplification*. In this pattern, multimodal monitoring capabilities were reduced to a single perceived function. During the observation period, one older adult (male, age 72) in Village B consistently treated an intelligent voice blood pressure monitor (which was equipped with a 4G Cat.1 voice module and supported one-sentence wake-up, full-process voice guidance, AI-powered interpretation, and cloud-based data synchronization) as a simple one-button measurement device. In interviews, he explained that he relied only on the numerical readout and deliberately avoided the voice interaction features. He explained: “I have to say so many things just to measure blood pressure. I am afraid the device won’t understand my dialect if I say something wrong. Just reading the number is enough. These fancy features are a burden for me.” This tendency toward simplification illustrates a cognitive defense mechanism in response to perceived technological complexity.

*Type 2: Functional misunderstanding.* This pattern involves an incorrect interpretation of technical functions that becomes reinforced over time. An older adult (female, age 68) in Village A misperceived the telemedicine terminal, which requires activating an online doctor transfer mechanism via a designated consultation button, as a conventional telephone that “connects directly to the doctor with one click”. Field records indicate that during an episode of acute gastric discomfort, she failed to obtain timely assistance. She mistakenly pressed the information inquiry button, having confused it with the consultation button due to their visual similarity. A technical support staff member (male, age 37) stated: “Even though we repeatedly emphasized key positioning in training, using color coding and shape differentiation as multisensory cues, older adults still have difficulty distinguishing between the buttons, especially in emergencies.”

*Type 3: Functional generalization*. This pattern occurs when users idealize equipment capabilities beyond their actual design limits. An older adult (female, age 75) in Village A expressed unrealistic expectations toward the fall detection device. The device had a detection range of 4 meters by 5 meters and consisted of a human fall monitoring radar, a Wi-Fi network, and a cloud-based management platform with real-time alert functionality. She asked: “If the system can detect falls, why can’t it automatically call for help and send a doctor to my home?” This expectation of integrated detection and rescue reflects a significant mismatch between perceived capabilities and the actual availability of emergency medical resources in rural areas. As a result, her overall satisfaction with the device was substantially reduced. A physician (male, age 42) at a local primary healthcare facility explained, “Although we repeatedly emphasize the functional limitations of these devices, older adults often expect an immediate medical response that exceeds what rural emergency services can realistically provide. This gap between expectations and reality has become a major barrier to technology acceptance.”

##### 3.2.1.2 Sub theme 2: procedural barriers to execution

Even when older adults in rural areas have developed a basic functional understanding of digital health technologies, many still face considerable difficulties in carrying out the required steps for operation. Prior research shows that complex user interfaces, multi-step operational sequences, and the lack of built-in error tolerance mechanisms constitute major barriers for this population (14). These procedural barriers not only reflect inadequate age-friendly design but also highlight a broader gap in technology developers’ understanding of the unique needs and usage habits of older adults living in rural settings.

A 79-year-old male participant in Village C demonstrated typical procedural difficulties when attempting to configure a smart pillbox. The setup process required six sequential steps: powering the device through charging or battery insertion, registering and linking the device to a mobile application, setting reminder parameters, placing medications into designated compartments, receiving scheduled alerts, and confirming medication intake. Field observations revealed that his first attempt failed. He did not press and hold the settings button for 3 s, which was the required condition to activate the device. During his second attempt, he successfully powered on the pillbox. However, he accidentally triggered the “dose memory” function, which reset all previously configured parameters. In the third attempt, he entered the wrong shortcut key combination for configuring reminders, which required simultaneous pressing of the “mode” and “time period” buttons. The configuration was ultimately completed with the assistance of a family member via video call, and the entire process took 42 min. The participant expressed frustration and self-doubt, stating, “The pillbox I used before just opened and worked. This one is far too complicated. I’ve spent so much time learning, and I still can’t do it. Maybe I’m just too old and useless now.”

Further observation indicates that execution barriers were significantly exacerbated by specific ergonomic micro-design flaws. During fieldwork, we noted that the smart pillbox used functional buttons measuring approximately 6 mm in diameter. The buttons were positioned too close together and offered no salient tactile cues or color coding to support discrimination. Such a configuration is particularly demanding for older adults with hand tremor or reduced fine motor control. Consequently, the participant repeatedly mis-pressed the reset button when attempting to confirm an action, which frequently interrupted the medication-taking sequence. Similar problems were reported by other users. For example, another older adult in Village C (female, age 76) Stated: “These small buttons are all crowded together. If my hand shakes even a little, I press the wrong one. I can’t tell them apart.”

Age-related declines in hand-eye coordination and visual function further intensify the operational challenges faced by older adults. An 83-year-old female participant in Village A, who experienced mild cataracts and hand tremors, struggled to operate the micro-lancing needle while using a smart blood glucose monitor. Due to instability during the sampling process, she failed multiple blood collection attempts, which resulted in finger pain and emotional distress. Furthermore, although the device was equipped with a 3.5-inch display screen, the default front size was approximately 12 points, and the interface utilized a black-on-light-gray color scheme. The contrast and visual settings did not align with older adults’ visual needs. As a result, she was unable to clearly read her glucose readings. During fieldwork, she repeatedly brought the device close to her eyes and kept changing the viewing angle. Even so, she could not identify the readings on her own and ultimately relied on family members to interpret them. As she said with resignation: “I can see a string of tiny lights, the numbers flickering there, but I just can’t make them out. Every time, I have to ask my daughter-in-law to read it for me.” This mismatch between age-related physiological decline and inadequately adapted device design represents a fundamental biological basis of the *usage divide*.

Dialect recognition failure in voice-interaction systems emerged as another salient barrier. In village B, an older adult (male, age 81) was provided with a “small wristband and gateway” package featuring voice interaction. In principle, this system allowed users to inquire about blood pressure, blood glucose, and medication reminders. Field observations, however, revealed that the speech recognition supported only standard Mandarin and had an extremely low recognition rate for the local dialect. Although the participant followed the training instructions, he repeatedly issued commands in his local dialect, such as “check today’s blood pressure.” The device either did not respond or returned irrelevant prompts, for example, “Unable to recognize your command, please try again.” After several failed attempts, he became frustrated and turned off the voice function entirely, using the wristband only as a regular watch. As he stated: “It can’t understand what I’m saying, and I can not be bothered talking to it anymore.”

##### 3.2.1.3 Sub theme 3: inadequate problem-solving strategies

Enhancing older adults’ ability to troubleshoot and solve problems, along with providing tailored instructional support, is essential for improving their effective use of digital health tools (5). However, rural older adults often lack the necessary strategies to respond to technical difficulties. This absence of problem-solving capacity is not limited to the technical level, but also reflects deeper differences in learning orientation and adaptation to unfamiliar digital environments.

A representative case involved a 70-year-old male participant in Village D. When his smart bracelet displayed a “network not connected” error, his first response was to restart the device, drawing on prior experience with household appliances. After this attempt failed, he adopted a passive waiting approach. He placed the device aside, hoping the issue would resolve itself over time. The problem remained unaddressed for 2 weeks, until it was eventually discovered and resolved by a community health worker during a routine home visit. During the interview, the participant shared: “I didn’t know what to do in this situation. I couldn’t understand the manual, and I felt embarrassed to keep asking others for help.”

This lack of strategic response can be traced to a fundamental generational gap in learning orientation. Unlike younger cohorts who developed digital literacy through iterative trial-and-error in technology-rich environments, rural older adults have neither the prior exposure nor the confidence to engage in exploratory learning.

A 78-year-old female participant in Village A shared her concerns about using a non-wearable sleep monitoring mattress. She explained: “The manual says it should be placed under the mattress to monitor sleep quality, heart rate, breathing, and movement. But I’m afraid to press the crescent-shaped buttons used to switch modes. What if I press the wrong one and damage the device? Our generation was taught to take care of things and not to touch unfamiliar objects without knowing how they work. And when something goes wrong, we don’t even know who to ask for help.” The community health worker responsible for training (female, age 36) further observed: “Even when we provide illustrated instructions and real-time practice opportunities, older adults often lack the basic skills to troubleshoot technical issues. This highlights the need for long-term capacity-building efforts, which cannot be addressed through one-time training sessions alone.”

#### 3.2.2 *Motivation divide*: from “unwilling to use” to “afraid to use”

The *motivation divide* refers to systemic disparities in the adoption and continued use of digital health technologies, arising from insufficient motivational factors (37). These factors are often reflected in reluctance to engage, only superficial use, or even the abandonment of essential device functions. Research has consistently shown that perceived usefulness and perceived ease of use are critical determinants of older adults’ intention and actual behaviors regarding technology adoption (38). Compared with the *skills divide*, motivational barriers are often less visible, yet they represent a more fundamental obstacle that can prevent the realization of the intended benefits of digital technologies.

##### 3.2.2.1 Sub theme 1: intergenerational gaps in value perception

The value perception of digital health technologies among rural older adults is deeply influenced by traditional health beliefs and longstanding cultural values, leading to a pronounced misalignment with the value propositions assumed by technology designers (39). Many participants expressed a passive health orientation, characterized by the belief that medical help is only necessary when physical discomfort arises. This orientation reflects an entrenched belief system that limits their appreciation for the potential benefits of preventive monitoring and proactive health management.

An 86-year-old male resident in Village E exemplifies resistance toward the anti-wandering wristband. He stated, “My mind is perfectly clear and my memory is good; what purpose does this thing serve at home?” The device incorporates GPS, Beidou, Wi-Fi, and accelerometer technologies to enable hybrid indoor-outdoor positioning. It is equipped with an electronic fence feature that provides real-time location tracking and triggers automatic alerts if the user crosses preset boundaries. Although family members repeatedly explained the bracelet’s preventive function, the older adult wore it only occasionally under his or her supervision. He never used it voluntarily, and no self-initiated activations were recorded during the month. He also demanded that the positioning function be disabled, expressing his frustration: “Do I really have to be monitored inside my own home? Am I a child or a criminal? I have lived this long, so why would I need a machine to tell me how to live?”

This divergence in value perception further extends to fundamentally different understandings of the nature of family-based care. Within the framework of traditional Chinese filial piety, emotional fulfillment is strongly associated with direct, in-person caregiving by children. Consequently, technology-mediated forms of remote assistance are often regarded not as a meaningful enhancement but as a substitute.

A 79-year-old female participant from Village B described her experience with an emergency call system installed in her bedroom: “The government installed a one-button call device and said it could be used for emergencies.” Although she understood its intended function, she questioned its emotional adequacy. She explained, “How can this compare to a real person? No matter how intelligent a machine is, it cannot sense how uncomfortable I feel. It is more reassuring to go to the village committee, where at least I can talk to someone and ease my loneliness.” A 34-year-old female nurse at a primary healthcare facility further commented, “Even though we demonstrated the system’s linkage features and emergency response speed during health education sessions, older adults still tend to trust the emotional support of face-to-face interactions more. Their acceptance of technology-based services remains generally low.”

##### 3.2.2.2 Sub theme 2: multiple manifestations of psychological resistance

Psychological factors constitute a major barrier to both the adoption and continued use of digital health technologies among older adults (40). Based on intensive field observations and in-depth interviews, this study identified four interrelated types of psychological barriers. These barriers capture the complex process of psychological adjustment that older adults experience when confronted with technological change.

Among them, the fear of causing damage emerged as the most common and consequential form of resistance. For instance, an 87-year-old woman in Village E persistently refused to operate a smart wheelchair on her own, despite repeated demonstrations by her family. The device, valued at more than 5,000 Chinese yuan (CNY), was equipped with advanced features such as electric drive, automatic obstacle avoidance, and remote control. She explained, “My eyesight is poor, and my hands are not steady. What if I press the wrong button and break it? Such an expensive machine is beyond what I can afford to replace. I would rather rely on my old wheelchair and feel at ease.” This fear was not only rooted in financial concerns; it also revealed her deeper anxiety over her declining physical and cognitive abilities.

Privacy and security concerns are particularly salient for devices that record health data. They highlight the instinctive vigilance older adults display toward personal information protection in the digital era. A 73-year-old male resident in Village C refused to use a terminal with an electronic health record function. He explained: “If my medication history and blood pressure records are uploaded online, my privacy will be completely exposed. What if someone exploits this information?” Despite the device’s use of end-to-end encryption and its compliance with the *Personal Information Protection Law of the People’s Republic of China*, and despite repeated explanations by technical staff, he remained cautious and ultimately decided not to use the feature.

Technology dependence anxiety reflects a deeper concern about losing autonomy. The ambivalence of a 78-year-old woman in Village B is telling. She stated: “If I become used to these devices, and one day they break down or the power goes out, will I be left helpless? As people grow old, they are already in decline. If we depend too much on machines, we may eventually lose even the basic ability to live.” This anxiety underscores the trade-off older adults perceive between technological convenience and the preservation of personal capability.

Concerns about technical accuracy arose when frequent false alarms undermined trust. During the second observation period, fall monitors often misinterpreted bending motions, such as picking up objects, as falls. Several families chose to deactivate the device. A 69-year-old man in Village D voiced his frustration: “Every time the alarm sounded, the community office called, which made the whole household nervous. Now I hesitate even to bend down while sweeping. Is this help or disturbance?” A 29-year-old technical support staff admitted: “Adjusting the sensitivity threshold can reduce false alarms, but in rural areas the limited coverage of professional technical support makes it difficult to address such issues promptly.”

##### 3.2.2.3 Sub theme 3: persistent low self-efficacy

Repeated setbacks in using digital health technologies can significantly reduce older adults’ technological self-efficacy, creating a vicious cycle described as failure-withdrawal-further failure (41). Field observations revealed that accumulated micro setbacks, such as device pairing failures, unstable synchronization, complex interfaces, and false or missed alarms, often led to learned helplessness and technological fatigue.

The experience of a 74-year-old male from Village C demonstrates this trajectory. At first, he showed strong motivation to adopt the smart bracelet, attending training sessions and exploring its functions. Yet a series of operational difficulties gradually eroded his confidence. The touch screen lacked sensitivity, causing frequent mistouches. Unstable connections repeatedly interrupted data synchronization. The emergency button was accidentally pressed multiple times, producing false alarms. After one incident in which community staff made a late-night visit to address a false alert, his attitude changed completely. He stated: “I’m too clumsy to learn these high-tech devices. I won’t use them anymore, or I’ll only create more trouble for others.”

Social comparison further intensified this loss of self-efficacy. A 66-year-old man in Village B contrasted his experience with that of his neighbor using an intelligent voice assistant. The device supported three preset emergency contacts, voice commands, and health reminders. He reflected: “My neighbor can set up three emergency numbers, make calls by pressing the emergency button, and even ask about the weather. I can barely use any of these functions except for a few accidental activations. People say I am like rotten wood that cannot be carved, and perhaps they are right.” Such self-deprecating judgments, rooted in social comparison, not only undermine individual adoption but may also generate negative collective perceptions within the community.

#### 3.2.3 *Support divide*: from “no one teaches” to “cannot teach effectively”

The *support divide* highlights how insufficient external support systems restrict the effectiveness of technology adoption. Even when devices and network access are available, the absence of accessible, comprehensible, and sustainable technical support often results in ineffective use and diminished benefits (42). In rural China, the organization and implementation of age-friendly home modifications depend largely on administrative mobilization by government departments and the promotion of special projects. Non-governmental actors, however, demonstrate weak implementation capacity. As a result, it remains difficult to establish a continuous system of operational maintenance and professional services. This structural deficiency directly manifests as a *support divide*.

This study finds that, in the context of age-friendly home modifications in rural China, stakeholders hold differing views on who should be responsible for technology use. These differences repeatedly surfaced in day-to-day support practices and were further reinforced through routine interactions.

##### 3.2.3.1 Sub theme 1: the formalization dilemma in formal training

Although China’s policy framework for age-friendly home modifications includes a training component, the actual outcomes fall short of the intended objectives. Effective use of digital health technologies requires repeated reinforcement, individualized guidance, and sustained follow-up (5). However, current delivery models are systematically deficient along these dimensions.

Field observations point to a threefold formalization dilemma. First, there is a mismatch between training duration and content depth. Instruction is typically provided orally by installation personnel at the time of setup and lasts about two to 3 h. Within this window, trainers must explain and demonstrate the functions of three to five devices, leaving very limited time per function (often 15–20 min). Consequently, demonstrations are cursory, with little opportunity for hands-on practice or feedback.

Second, the language of instruction exceeds the cognitive threshold of many older adults. Although most participants possess only basic literacy, the training materials are saturated with technical terms such as “Bluetooth pairing,” “cloud synchronization,” and “threshold setting,” which are difficult to interpret without scaffolding or plain-language glosses. This disconnect raises the entry barrier and undermines confidence.

Third, post-training follow-up is largely absent. Sessions are commonly treated as a one-off service, with no structured refresher training, outcome monitoring, or mechanisms to identify and address persistent use problems over time. In combination, these shortcomings weaken skill acquisition and impede the sustained use of installed technologies.

An account from a primary-level community worker (male, age 41) illustrates how training is actually implemented: “To be honest, training is mostly about completing tasks assigned from above and taking photos for the record. We have little idea how much older adults remember or use afterward. Since the higher authorities require training records, we organize sessions, take attendance, and take pictures, but no one really evaluates the outcomes.” Feedback from older participants corroborates this tendency toward formalization. Many report that they “forget soon after the training”, and only a few describe it as “somewhat helpful, but insufficient”.

Moreover, the mismatch between training content and real-world needs further undermines effectiveness. Standardized sessions ignore individual differences in vision, hearing, cognitive ability, and learning pace, and they rely on a uniform instructional model. As one older adult (male, age 71) from Village C put it, “The training is like teaching in English to people who only understand the local dialect; it sounds like gibberish to us. What seems simple to younger people feels almost impossible for us.”

Clear attributional divergences emerged across implementation actors when explaining why training was often ineffective. Frontline community workers tended to frame the problem as a consequence of policy compliance pressures and resource constrains. As one community staff (female, age 38) noted: “There are deadlines from above, and we have to hit the coverage targets by a certain date. Training matters, sure, but what they check is the numbers. We do not really have a choice.” In contrast, technical support personnel responsible for device installation and troubleshooting more often attributed the problem to design deficiencies in the device themselves: “These devices are not made for older adults. You can train them all you want; it still will not work. If the interface stays the same, they will not remember all these steps. This is on the tech companies, not on us.” This divergence between institutional attribution and design-based attribution created a shift in perceived accountability. In everyday practice, responsibility for training was repeatedly pushed outward and reassigned to other parties, rather than being treated as a shared obligation within the implementation chain.

##### 3.2.3.2 Sub theme 2: systemic gaps in family support

Families are ideally positioned to support older adults in adopting digital health technologies, yet widespread empty-nest patterns in rural China have created a support vacuum (43). In our sample of 24 households, 19 (79.2%) had adult children working away from home year-round and returning, on average, for fewer than 30 days each year. Even when children are present, intergenerational reverse mentoring in the digital domain encounters multiple, layered obstacles.

Insufficient time investment is the primary constraint. Family caregivers frequently report that technology instruction takes far longer than expected. One explained (male, age 42), “My parents learn very slowly; the same operation has to be repeated dozens of times before it barely sticks.” Another added (male, age 34), “Work pressure is high, time at home is limited, and our patience is in short supply.” Field observations indicate that a typical teaching interaction by adult children lasts about 10 min on average, which is far less than the time older adults generally need to consolidate new skills. This substantial gap in instructional time directly undermines learning outcomes.

Ineffective teaching methods further erode the value of family support. There is a marked mismatch between the rapid demonstration style favored by younger adults and the cognitive processing speed of older adults. For instance, one older adult (female, age 79) in Village B described her son completing the entire setup of a smart pillbox in 3 min: power on, select mode, set time periods, confirm dose. She recalled, “His fingers moved quickly over the small buttons. Before I could even locate the confirmation key, all three medication periods had been set. When I try to do it on my own, I cannot remember the first step.” This mismatch between fast input and slow processing constitutes a key barrier to intergenerational technology transfer.

A clear positional divide emerged between family caregivers and frontline community workers over who should take primary responsibility for household support. Family caregivers tended to push this responsibility toward community services and technology providers. As one adult child who worked away from-home (male, age 34) said: “I only come back for a few days a year. I can’t be there every day to keep teaching them. If the government is promoting these devices, there should be ongoing technical support. You can’t just install them and then walk away.” In contrast, frontline community staff argued that families should be the first line of support. A village committee member (female, age 45) responded: “We don’t have enough staff. We can’t be a ‘tech babysitter’ for every older adult. Their children know them best, and they should make time to teach their parents.” This disagreement, shaped by competing claims about time constraints and where responsibility should lie, left household support in a prolonged state of limbo in everyday practice.

Emotional factors create a deeper, often overlooked barrier. Many older adults refrain from seeking help out of a traditional reluctance to impose on their children, which produces a dilemma of wanting to learn but not daring to ask. One older adult (female, age 77) in Village A stated, “It is not easy for children who work away from home, and their job pressure is high. I do not want to trouble them with such a small matter. I will try to figure it out slowly, and if I still cannot use it, so be it.” Her family caregiver (female, age 52) offered the other side of this dynamic: “Each time I teach her a new function, she says she can manage because she does not want to take up my time. Yet when she actually needs it, she still cannot use it, and the cycle repeats.”

##### 3.2.3.3 Sub theme 3: resource bottlenecks in community-based support

Community-level technical support is constrained by scarce resources and limited capacity. Within China’s primary healthcare system, staffing for aging-in-place services is critically inadequate. The shortfall spans both community staff and specialized technical personnel, and prevailing ratios remain well below those reported in high-income countries (44). This resource deficit translates into coverage gaps in support services, leaving many households without timely assistance.

Geographic accessibility markedly affects the effectiveness of support service. Remote villages located more than five kilometers from town centers face average response times of five to 7 days for technical support, whereas suburban villages typically wait only one to 3 days. These spatial disparities widen the *usage divide* within rural areas. A technical support worker (male, age 37) acknowledged the staffing shortage: “There are only a few technicians for the entire district, and we are responsible for maintaining age-friendly equipment across hundreds of villages. We must prioritize hardware failures, and most issues related to user guidance cannot be addressed promptly because we simply lack the personnel.”

Professional capacity limitations also undermine the quality of community support. Only a small share of frontline staff in primary-level community organizations have received systematic training in digital health technologies, and most instead rely on self-study or brief orientations. A village committee cadre (male, age 53) acknowledged the limits of his competence, “Some advanced devices, such as the AI health butler that integrates natural language processing and machine learning, are only partially understood even by us. We end up learning while teaching. When older adults ask more detailed questions, we cannot provide satisfactory answers.” This mode of support is unlikely to produce substantive results.

Resource-constrained work settings also shaped how community-based support actors assigned responsibility for using digital health technologies. A technical support personnel (male, age 29) member stressed design accountability, stating: “Tech companies should think about what older adults need when they design the product. They should not dump the age-friendly part on people like us at the very end.” In contrast, primary healthcare providers were more likely to attribute the difficulties to older adults’ limited digital literacy and entrenched health practices. As one village doctor (male, age 56) put it: “It’s not that older adults can’t learn. A lot of them just don’t want to, or they can’t be bothered. They think, ‘I’ve lived most of my life without this stuff and I was fine,’ so they reject it from the start. And some can barely read. What are we supposed to do then?” Although these attributions seem to point in opposite directions, they reflect a shared pattern of defensive boundary setting. Under chronic resource shortages and limited support capacity, different actors drew clear lines around what they saw as their own responsibilities and what they refused to carry.

#### 3.2.4 *Persistence divide*: from “temporary use” to “sustained use”

The *persistence divide* refers to a systemic disparity in the dynamic adaptability required to maintain effective technology use over time after initial device acquisition and network connectivity (45). This gap means that even individuals who possess the necessary devices and connectivity may struggle to continue using technologies in a stable and beneficial manner. It captures the complex shift from initial adoption to full integration into everyday life.

##### 3.2.4.1 Sub theme 1: barriers to forming stable technology habits

The formation of stable technology habits depends on repeated reinforcement and a supportive environment. Evidence suggests that turning a new behavior into an automatic habit typically requires about 66 days of consistent practice (46). By contrast, our field observations reveal a cliff-like decline in the use of digital health technologies among older adults in rural areas.

The absence of environmental triggers is a primary obstacle to habit formation. Unlike smartphones and other technologies with clear usage contexts, digital health devices rarely offer natural cues that prompt action. A 74-year-old man in Village C illustrated the point: “When the phone rings, you answer. When a television program starts, you watch. These actions are woven into daily life. By contrast, health devices, though important, are easily forgotten without reminders. They sit unused like decorations and gradually gather dust.”

Competition with established routines further limits the uptake of new technologies. Field observations show that although virtual community terminals capable of supporting real-time video interaction for up to 20 participants were available, most older adults still preferred in-person contact when seeking social engagement. In the morning exercise area in Village B, many chose to walk 10 min to join in square dancing rather than use the devices for online health exercises. One older adult (female, age 68) from Village B explained: “Online communication always feels like there is a barrier. You cannot see a real person or feel warmth. At our age, what we seek is human presence, and no machine, however advanced, can replace it.”

##### 3.2.4.2 Sub theme 2: difficulties adapting to system updates

A fundamental tension exists between the rapid iteration of digital technologies and the slower learning pace of older adults. During the observation period, several digital health devices received one or two system updates. Each update introduced interface changes, reorganized functions, or altered operating steps. For older users, this moving target creates an ongoing challenge, as procedures they had already learned become outdated and must be relearned.

The experience of a woman from Village D, age 67, illustrates the learning burden created by system updates: “I had finally learned how to check blood pressure and heart rate and could identify what each button did. After the update, the entire interface changed, the icons moved, and even the colors were different. Is this not making things difficult for older people like us?” Such skill resetting increases the cost of learning and, more importantly, erodes users’ confidence.

System updates often produce a pronounced skill depreciation effect among older adults. One resident (male, age 70) from Village B had mastered the original three-step video consultation on the telemedicine device. The sequence consisted of power on, select a doctor and initiate the call. After a subsequent optimization, the procedure expanded to five steps with the addition of identity verification and symptom prefilling. He expressed his frustration: “Everything I learned feels wasted, and I have to start over. At my age, how can I keep relearning again and again? Younger people may call it an optimization, but for us it often turns the system into something we can no longer use.”

##### 3.2.4.3 Sub theme 3: cumulative effects of repeated setbacks

Repeated setbacks during use have a cumulative effect that gradually erodes willingness to engage with the technology and eventually leads to abandonment. Field observations reveal a clear pattern. A single major setback, such as a failure to obtain emergency assistance, prompts most older adults to suspend use for at least 1 week. A series of minor setbacks, including operational errors or connection failures, then drives down the frequency of use. Once accumulated frustration exceeds an individual’s tolerance threshold, discontinuation becomes the most common outcome.

The emotional impact of frustration often generalizes beyond a single device, shaping perceptions of digital technologies as a whole. In Village B, a 64-year-old woman, after repeatedly failing to set up a smart pillbox, voiced deep self-doubt: “Am I too stupid to learn new things at this age?” Such negative self-attribution can prompt social withdrawal, as some older adults become reluctant to expose perceived incompetence. In a minority of cases, these reactions persist as low mood and feelings of worthlessness, further discouraging engagement with technology.

The absence of positive feedback mechanisms significantly accelerates abandonment. When usage fails to produce immediate, perceptible benefits, motivation declines rapidly. A family caregiver (female, age 49) described her father’s experience with a smart blood pressure monitor: “The device showed only the raw reading and read it aloud, without indicating whether the value was normal or offering personalized advice. After a month, my father said, ‘I cannot tell if this is good or bad, so the device is useless,” and he stopped using it.” This perceived imbalance between effort invested and benefits received directly undermines the willingness to continue using the technology.

## 4 Discussion

China’s population is aging at an accelerating pace, and the challenge is especially acute in rural areas. Improving the quality of elder care in these settings has therefore become a widely recognized priority. Within the current care system, rural older adults rely mainly on family-based care, an arrangement that accords with long-standing cultural values as well as the practical constraints of rural resources (7). Against this backdrop, age-friendly home modifications have emerged as a foundational strategy for improving the quality of older adult care.

In recent years, digital health technologies have expanded rapidly as a core enabling tool, and the urban–rural gap in infrastructure access has narrowed (47). Nevertheless, evidence shows that their health benefits remain limited by a *usage divide*, namely, barriers that persist after physical access is achieved (16). In rural China, this divide is particularly pronounced and substantially constrains the inclusive potential of digital health technologies. Accordingly, this study examines how the *usage divide* shapes technology use within age-friendly home modifications among rural older adults in China.

Through systematic inquiry, this study identified a *usage divide* in digital health technology among rural older adults in China, unfolding across four interlinked dimensions: the *skills divide*, *motivation divide*, *support divide*, and *persistence divide*. This perspective moves beyond the common tendency to attribute adoption barriers solely to skill deficits and aligns with the international three-level *digital divide* framework, while displaying features specific to the Chinese rural context (14). Unlike the emphasis in many developed settings on the transition from access to use (17), rural older adults in China encounter a more complex sequence of ruptures across access, use, and outcomes. Even where physical access has been achieved, multilayered challenges persist, a pattern also observed in rural areas of other developing countries (43). These findings deepen theoretical understanding of the *digital divide* and provide an empirical basis for designing targeted interventions.

Although the *skills divide* constitutes a fundamental barrier, the underlying mechanisms are far more intricate than a simple can-or-cannot dichotomy. Even after training, rural older adults continue to struggle at three progressive levels: functional understanding, operational execution, and problem solving. International evidence suggests that effective training for older adults often requires 60–90 min of repeated practice (44), yet actual training time in rural China falls well below this benchmark. These difficulties arise not only from individual cognitive differences but also from a mismatch between the design logics of digital health technologies and the mental models of older adults.

Field observations from this study further suggest that older adults’ reported inability to “use” digital health technologies often reflects age-related functional decline interacting with a systematic mismatch in design. On smart pillboxes, small buttons and complex multi-key operations made accurate input difficult for older adults with hand tremor or reduced fine motor control. Prior work indicates that a button size of around 20 mm is preferred by older adults and can be operated more reliably under conditions of motor decline (48). Yet many commercially available devices still fall short of this basic requirement.

A similar pattern was observed in smart blood glucose meters. Default interfaces commonly used small fonts and low-contrast displays, which limited independent reading for older adults with cataracts or vision loss. Age-friendly interface guidance recommends a minimum font size of 14 pt., and some studies suggest 18 to 22 pt. to accommodate declining visual function (49). It also recommends a contrast ratio of at least 4.5:1 (50). Meanwhile, voice interaction systems that did not support local dialects led to repeated recognition failures among rural older adults who rely on dialect in everyday communication. Although speech recognition tailored to older users and dialect speakers is advancing, such capabilities remain uncommon in consumer digital health devices.

Taken together, what is often labeled as “not knowing how to use” digital health technologies should not be treated as a simple learning failure or an individual capability deficit. Instead, it reflects a situated process in which older adults’ visual, motor, and linguistic characteristics are excluded from the assumptions built into the technology’s “default user.” In this sense, the *skills divide* is not located only on the side of older adults. It is embedded in the design logic of digital health technologies themselves. When systems are developed for an implicit user who is young, healthy, and a standard-language speaker, rural older adults are placed at a disadvantage at the point of task execution. This finding suggests that narrowing the skills divide cannot be achieved through more intensive training alone. It also requires rethinking age-friendly design upstream, so that digital health technologies align with older adults’ bodily capacities and everyday living contexts.

Lower levels of educational attainment in rural populations further exacerbate the *Skills Divide* (46). Equally crucial, the *motivation divide* highlights willingness as the pivot of adoption. Divergent value perceptions, psychological resistance, and low self-efficacy form an invisible barrier. Consequently, even with basic operational skills, many older adults refrain from using these technologies. This pattern is consistent with TAM and UTAUT, which emphasize perceived usefulness and perceived ease of use as decisive factors in technology acceptance (47).

The *support divide* and *persistence divide* highlight that technology use is both socially embedded and temporally fragile. On the social side, formalistic training, lack of family support, and severe shortages of community resources leave daily scaffolding weak. Over time, mismatches between the rapid pace of technological updates and the slower learning rhythms of older adults further erode continuity. Taken together, these conditions create an ecosystem that discourages sustained engagement. The four dimensions reinforce one another, generating a vicious cycle in which weak skills undermine motivation, inadequate support accelerates skill attrition, and cumulative frustration leads to eventual abandonment.

The role of culture in shaping the *usage divide* is substantial and provides non-Western empirical support for technology acceptance theory. Traditional Chinese values influence technology use among rural older adults through three pathways, and the pattern differs from findings reported in other Asian contexts (51). First, filial piety frames the evaluative logic of care. Many older adults regard technology-mediated remote care as lacking a human touch, a judgment rooted in a Confucian preference for emotionally grounded interaction. This orientation directly shapes perceived usefulness, a core construct in the Technology Acceptance Model (52). As a result, even when digital health tools perform effectively, they may be judged as having limited value if they do not accord with culturally expected modes of care. By contrast, older adults in many Western settings place greater emphasis on the independence and autonomy afforded by technology (53). This contrast illustrates how culture defines the normative criteria for what counts as good care, thereby guiding acceptance.

Second, a culture of thrift, combined with the norm of not troubling others, heightens anxiety around technology use. The dictum “cherish things; do not touch what you do not understand” clashes with the exploratory learning required in the digital era. Fear of damage arises not only from financial concerns but also from moral unease about violating frugality as a virtue (36). A widespread reluctance to seek help from one’s children further leads many older adults to endure difficulties alone, directly limiting access to necessary support (54). In contrast, Western cultural contexts more often encourage active help-seeking among older adults (55).

Third, face culture shapes self-efficacy through social comparison. In collectivist settings, technological competence has become a new axis of social evaluation (14). Several participants described feelings of shame after operational failures, noting that concern about losing face discouraged them from using technologies in public and reduced opportunities for practice and improvement. Similar dynamics have been documented in other East Asian contexts (52), although they appear less prevalent in predominantly individualist Western societies (56). Identifying these cultural mechanisms enriches explanations of technology acceptance and indicates that cultural fit should be treated as a design and implementation priority when promoting digital health tools.

An in-depth analysis of the *support divide* reveals systemic obstacles to the promotion of digital health technologies in rural China. These obstacles involve both quantitative shortfalls in resource allocation and qualitative deficiencies in the support that is actually delivered. The weaknesses of formal training exemplify the large gap between policy implementation and lived needs. In practice, training sessions are far shorter than international recommendations, which suggest 60 to 90 min of repeated practice for each function (51). Compared with systematic digital-literacy programs in high-income settings such as Canada (21), provision in rural China is markedly fragmented. Moreover, training is still heavily technology-centered and insufficiently adapted to older adults’ cognitive profiles. Dense technical terminology, limited accommodation for local dialects, and minimal attention to individual differences all reduce instructional effectiveness. More critically, training often becomes an administrative exercise aimed at fulfilling reporting requirements, with little attention to outcome evaluation or sustained follow-up. This formalism not only wastes scarce resources but, by delivering ineffective instruction, can also heighten technology-related anxiety among older adults.

Based on interviews with multiple types of supplementary informants, this study identified differentiated attribution patterns regarding the causes of the *support divide*. Community workers most often framed the problem in terms of policy compliance pressures and performance metrics that prioritized rapid coverage over sustained support. Technical support personnel, in contrast, located the cause at the product level and pointed to inadequate age-friendly design. Primary healthcare providers more frequently attributed the gap to older users’ limited digital literacy and weak willingness to engage with technology. Family caregivers, constrained by time and distance, emphasized that community organizations and technology firms should assume greater responsibility for ongoing support. These attributions should not be read as mere differences in personal opinion. Rather, they reflect role-based interpretations shaped by each actor’s institutional position, available resources, and professional responsibilities within the implementation process.

This finding aligns with international scholarship on barriers to digital health adoption. Prior research shows that implementation challenges span multiple levels, including technological, organizational, economic, and user-related domains (57). It is also suggested that actors in different roles tend to define problem boundaries and allocate responsibility based on what they can realistically control. Building on this literature, our ethnographic evidence indicates that when differentiated perspectives are not linked through an integration mechanism, support responsibility can easily shift from one party to another and eventually solidify into an institutionalized state of “no one is responsible.” Sustained implementation of digital health technologies requires coordinated governance and durable commitments across government agencies, service organizations, and other stakeholders. However, such institutionalized frameworks for multi-actor collaboration have yet to be established in rural China. Clarifying responsibilities and building cross-actor coordination are therefore central to embedding digital health technologies in older adults’ everyday lives and narrowing the *support divide*.

The absence of family support constitutes another major barrier. Although this challenge is global, it is particularly acute in rural China. The widespread empty-nesting phenomenon, with 45.4% of adult children living away year-round (58), disrupts the chain of intergenerational technology transmission. This pattern differs from many Western countries, where family support gaps arise primarily from geographical distance rather than demographic out-migration (59). Even during brief reunions, rapid demonstration styles are often mismatched to the slower cognitive tempo of older adults. The result is a dissonance between high-speed input and low-speed processing that undermines both retention and confidence (60). Evidence suggests that successful intergenerational transfer requires patience, repetition, and emotional reinforcement (54). However, these are precisely the elements that empty-nest families tend to lack. A cultural preference for not troubling one’s children further narrows the practical window for assistance. At the community level, resource bottlenecks reveal deeper limitations of the rural public service system. Ratios of technical staff to service recipients are severely imbalanced. Professional training is inadequate, and geographic accessibility varies markedly. Together these conditions create significant blind spots in support provision. Compared with high-income countries, rural China continues to face a substantial gap in building an age-friendly ecosystem for technological support (44).

The identification of the *Persistence Divide* highlights the barrier between short-term uptake and long-term integration. Research suggests that forming a stable technology-use habit requires about 66 days of sustained practice (46). Yet our fieldwork revealed a sharp decline: by around 1 month, most rural older adults had largely stopped using the tools. This rapid disengagement was more pronounced than among urban counterparts (14), pointing to additional, context-specific hurdles to habit formation in rural settings. Several mechanisms converge to impede routine use: the absence of environmental trigger cues, competition with entrenched daily routines, skill depreciation caused by frequent product updates, and the cumulative effects of repeated setbacks. The tension between the pace of technological change and the slower learning rhythms of older adults is especially acute in rural areas, where ongoing learning support and update guidance are scarce.

Although this study focuses on the *usage divide*, its findings also shed light on the *outcome divide*. Translating the health-promotion potential of digital health tools into tangible gains presupposes stable and meaningful use. Yet among rural older adults in China, there is a pronounced break in the sequence from access to use to outcomes. This rupture takes the form of a dual differentiation. Older adults with higher educational attainment and adequate family support are more likely to overcome usage barriers and become part of a digitally advantaged group. By contrast, those with limited education, solitary living arrangements, and heavy chronic disease burdens often face adoption difficulties that lead to secondary marginalization. The resulting inequities constrain the inclusive value of these technologies and risk widening health disparities, thereby generating new layers of inequality in health outcomes.

Many of the observed *usage divide*s originate from a persistent misalignment between technology design and the actual capabilities and needs of rural older adults. Contemporary digital health tools often display pronounced function stacking: developers prioritize novelty and specification gains while neglecting users’ contexts of use and their usability constraints. This philosophy runs counter to rural older adults’ preference for solutions that are simple, intuitive, and stable. International experience indicates that successful tools for older users adhere to principles of parsimonious or simple design (61), yet this standard remains under-implemented in China’s rural age-friendly renovations. The findings therefore call for a fundamental shift in design practice. The field must pivot from technology-led to user-led development, from feature-first to experience-first decision-making, and from rapid iteration to stability and reliability. The overarching objective is to move beyond mere technological access toward genuine health empowerment by improving design fit. Achieving this shift requires not only technical innovation but also coordinated action across policy, market, and community sectors to build a rural digital health ecosystem that is truly centered on older adults.

## 5 Strengths and limitations

This study offers two key strengths. First, it employs a focused ethnographic approach combined with grounded theory, which allowed for in-depth exploration of the usage divide as embedded in the daily lives and cultural contexts of rural older adults. This methodological depth addresses the gap in existing literature that often relies on quantitative surveys with limited contextual sensitivity. Second, by centering on rural China, a setting underrepresented in digital health research, it provides unique empirical insights into how structural constraints (e.g., empty-nest families, fragmented support systems) and cultural values (e.g., filial piety, face culture) shape technology use, thereby enriching the global understanding of digital inequality in aging populations.

This study has several limitations. First, the sample has limited representativeness, as all fieldwork was conducted in Bishan District, Chongqing. The transferability of the findings is therefore constrained. Future research should broaden the geographic scope to include rural areas with different levels of economic development and diverse cultural contexts. Second, the ethnographic approach carries inherent subjectivity. Despite employing multiple credibility-enhancing procedures, the researcher’s positionality as a technical outsider may have shaped both observation and interpretation. Mixed-methods designs that triangulate qualitative insights with quantitative data would help strengthen the robustness of inference. Third, the absence of true longitudinal tracking restricts insight into the long-term evolution of technology use. Although three rounds of observation were conducted, they were insufficient to capture full trajectories of adoption and attrition. Prospective studies spanning 2 to 3 years would provide a clearer picture of the dynamic mechanisms underlying the *usage divide*. Finally, this was an observational study and did not test interventions. As a result, the effectiveness of candidate strategies for narrowing the *usage divide* remains unknown. Subsequent research should design, implement, and evaluate targeted interventions to determine real-world impact.

## 6 Conclusion

Using an ethnographic approach, this study illuminated the complex nature of the *usage divide* in digital health technology among older adults aging in place in rural China. The findings indicate that the divide is not merely a matter of skills but also reflects systemic challenges of motivation, support, and persistence. Cultural values, social structures, and design choices interact to shape patterns of both use and non-use. Closing the *usage divide* requires moving beyond technological determinism toward an integrated approach that aligns technology, individuals, and environments. Progress depends on coordinated action across multiple levels and actors, including designers, policymakers, service providers, communities, and families. Only through such multi-level, multi-stakeholder collaboration can digital health technologies achieve broad and equitable uptake among rural older adults, thereby advancing the dual objectives of healthy aging and digital inclusion.

## Data Availability

The original contributions presented in the study are included in the article/supplementary material, further inquiries can be directed to the corresponding author.
